# Case of Tuberculated Peritoneum

**Published:** 1826-07

**Authors:** 


6. *Diseased Peritoneum.* Dr. Crowther has lately related an interesting case of this kind in our Northern cotemporary. The patient was a young lady, aged 30, who, having been seized in December, 1823, with febrile symptoms, sickness, and pain in the lower part of the abdomen, was apparently cured by common means. But, in March following, she was found to be considerably emaciated, with dry skin, quick pulse, distention and pain in the abdomen. Fluctuation was evident there. Diuretics and mercurials produced no benefit. In April, 1824, when Dr. C. saw her, she appeared to have common ascites, and the usual remedies were prescribed without effect. She continued to increase in size, and was tapped in August, but only two quarts of a fluid resembling water-gruel were discharged. In another attempt, about four quarts were evacuated from a puncture in the linea alba. Hardness and inequality were now perceptible in the right side of the abdomen and the body was fast extenuating. She lingered in great distress till October, when she died.

*Dissection.* The trochar was pushed, with much difficulty, into different parts of the abdomen, and about half a gallon of thick fluid flowed out. The parietes were then dissected back, and the peritoneum laid bare. It was from one to two inches and a half in thickness, and white throughout, consisting of firmly condensed cellular membrane. "A heterogeneous mass presented itself, containing cysts of various forms and sizes, some round, some oval, some transparent, some opake, some capable of containing two quarts, others not half an ounce. The transparent cysts contained a thick, transparent, spongy, gelatinous fluid—the white cysts contained a white fluid, of the consistence of thick cream—the coats of some of the larger cysts were very thick and opaque—there likewise existed a quantity of empty cysts, and flakes of coagulated lymph. The whole had the appearance of an immense quantity of purulent matter, floating amidst the hydatid cysts between the laminæ of the peritoneum, and amounting to six or seven gallons." There was no adhesion between the peritoneum and the abdominal viscera, nor was any fluid found in the general cavity of the abdomen.

This was evidently a case of hydatid or tubercular disease, of which Dr. Baron has given the best description of any writer with which we are acquainted. We have at this moment a melancholy case of the kind under our care, in the person of an accomplished young lady from Scotland. The abdomen is enlarging, and partial fluctuation is perceptible. The rest of the body is gradually wasting away—and the sense of distention, especially after eating, is very distresssing. Death will, no doubt, close the scene. Repeated leeching is the only measure which gives a temporary relief.

